# The contribution of beef consumed as part of the National School Lunch Program to nutrient intake and adequacy: a National Health and Nutrition Examination Survey 2003–2018 Analysis

**DOI:** 10.1016/j.cdnut.2025.107603

**Published:** 2025-11-24

**Authors:** Kristina S Petersen, Kristin Fulgoni, Victor L Fulgoni

**Affiliations:** 1 Department of Nutritional Sciences, Pennsylvania State University, University Park, PA, United States; 2 Nutrition Impact LLC, Battle Creek, MI, United States

**Keywords:** beef, National School Lunch Program, nutrient intake, nutrient adequacy, red meat, meat

## Abstract

**Background:**

Beef intake in youth is associated with higher micronutrient intake. The contribution of beef consumed as part of the National School Lunch Program (NSLP) to nutrient intake is unclear, and warrants investigation given the variety of options used to meet the NSLP meats/meat alternates requirements.

**Objectives:**

The aim was to examine nutrient intake and adequacy in NSLP participants consuming beef, compared with those not consuming beef, overall, by gender–age categories, and food security status.

**Methods:**

Data from the National Health and Nutrition Examination Survey (NHANES) 2003–2018 were used. NSLP participants were defined by a previously published method. Nutrient intake was assessed from the first 24-h recall. Usual nutrient intake, calculated from both recalls, was used to estimate nutrient adequacy assessed by the percentage of the population below the estimated average requirement (EAR); percentage above the adequate intake (AI) was also estimated. Regression analyses were used to examine differences in nutrient intake between beef consumers compared with nonconsumers at school lunch with adjustment for age, sex, race/ethnicity, and energy intake.

**Results:**

The analytical sample included 3046 (weighted *n* = 18,787,280) NSLP participants, which included 1153 (*n* = 7,024,904) beef consumers and 1893 (*n* = 11,762,376) nonconsumers of beef at lunch. Overall, beef consumers at school lunch had a median beef intake of 14 g (IQR 5, 41). Beef consumers at school lunch had higher intakes of saturated fat (2.3 ± 0.5 g; *P* < 0.001), total fat (2.0 ± 1.0 g; *P* = 0.04), vitamin B12 (0.6 ± 0.2 μg; *P* = 0.003), and zinc (1.1 ± 0.3 mg; *P* < 0.001), and lower intake of carbohydrates (−6.2 ± 2.8 g; *P* = 0.03), niacin (−1.4 ± 0.4 mg; *P* = 0.002), and vitamin E (−0.5 ± 0.2 mg; *P* < 0.001) compared with beef nonconsumers at school lunch. A lower percentage of beef consumers at school lunch, compared with beef nonconsumers, had zinc intake (−3.94 ± 1.97 percentage points; *P* = 0.045) less than the EAR.

**Conclusions:**

Beef intake as part of the NSLP made a limited contribution to nutrient intake and adequacy; however, beef intake at school lunch was low.

## Introduction

The United States Department of Agriculture administers the National School Lunch Program (NSLP) to provide eligible students with a nutritious, low-cost or no cost lunch. Nearly all public schools and 94% of all schools (public and private) participate in the NSLP, resulting in ∼30 million students participating in the NSLP [1]. Prior research using the National Health and Nutrition Examination Survey (NHANES) data has shown that NSLP participation is associated with higher diet quality compared with nonparticipation [2,3]. Furthermore, lunches provided as part of the NSLP are more nutrient-dense than lunches brought from home [4,5]. This is because of the NSLP nutrition standards. In 2012, the NSLP nutrition standards were updated to align with the 2010 Dietary Guidelines for Americans [6]. Of note, daily and weekly requirements for minimum servings of meat/meat alternates, fruits, vegetables (including specific subgroups weekly), whole grains, and milk were introduced. In 2024, the nutrition standards were further updated so that beans, peas, and lentils provided as the meats/meat alternates meal component could also count toward the weekly requirement for the beans, peas, and lentils vegetable subgroup [7]. This change was made to support schools in providing more plant-based and vegetarian options.

In the NSLP, selection of the meats/meat alternates meal component is high, and plate wastage is low [8,9]. However, a study conducted in 2 middle schools in Georgia showed that plant-based entrees were selected at a much lower rate, and plate waste was significantly greater than meat-based entrees [10]. Other studies conducted in Florida and Maryland suggest that vegan [11] and soy-based [12] meal components are selected at a similar rate to meat-based meal components. Given the recent interest in incorporating more plant-based and vegetarian options into the NSLP for health and sustainability reasons, investigation of the contribution of meats, including commonly consumed meats such as beef, provided as part of NSLP meals to overall nutrient intake is needed.

Beef is a nutrient-dense food that contributes to nutrient intake in children and adolescents [13,14]. An analysis of NHANES data from 1999 to 2004 showed that beef intake contributed significantly to the overall intake of vitamins B6 and B12, zinc, iron, niacin, phosphorus, and potassium [13]. In a more recent analysis using NHANES 2001–2018, a lower percentage of adolescents (age 14 to 18 y) who consumed beef, compared with those who did not consume beef, had intakes below the estimated average requirement (EAR) for calcium, copper, folate, iron, phosphorus, riboflavin, thiamin, vitamin B6, vitamin B12, and zinc [14]. The contribution of beef consumed as part of the NSLP to nutrient adequacy has not been investigated. Thus, the aim of these analyses using NHANES 2003–2018 is to examine nutrient intake and nutrient adequacy in students consuming beef as part of the NSLP, compared with students consuming an NSLP meal without beef, overall and by age and gender category. We also conducted a subgroup analysis by food security status because food insecurity is associated with a higher risk of nutrient inadequacies in children [15].

## Methods

### Data source and study population

The NHANES is a government-run program used to continuously assess the nutrition and health status of the United States population using a nationally representative sample of noninstitutionalized individuals. The study population included those aged 5 to 18 y who participated in NHANES 2003–2018 cycles. Participants with unreliable dietary recall(s), zero energy reported, and pregnant and/or lactating females were removed from the sample, resulting in a final sample size of 19,482. The present study utilized the individual food files and total nutrient files from both What We Eat In America 24-h dietary recalls. Demographic variables were sourced from the NHANES demographics questionnaire. Food security was defined as food secure (full/marginal food security), low food security, and very low food security based on NHANES classifications of child food security. Beef consumers were defined as participants who consumed any beef. Further information on NHANES data and methodology can be found on the NHANES website [16,17].

### Beef intake calculations

Beef intake was determined in the same manner as previous publications [14,18]. In short, the Food and Nutrient Database for Dietary Studies was used in conjunction with both the Food Pattern Equivalents Database and the Food Patterns Ingredients Database (FPID). Ingredient codes from FPID that contained beef (defined as positive values for protein foods: meat and cured meat) were examined to determine the percentage of beef present. If the ingredient containing beef was <100% beef (i.e., hot dogs, bologna), a multiplication factor was utilized to quantify the amount of beef within the ingredient. Several food codes were missing FPID nutrient values or were modified food codes, such as reduced sodium. These missing ingredient profiles were replaced with complete profiles from the same food code of previous NHANES cycles, or the ingredient was replaced with the nutrient profile of a similar, hand-matched ingredient.

### School meal participants

NSLP participation was defined according to methods previously used [2,3] because NHANES does not include questions assessing NSLP participation. NSLP participants were defined as reporting a lunch meal between 10:00 and 14:00 and one of the following:

1). Consuming 3 school lunch components sourced from a school cafeteria,
2). Consuming 2 school lunch components sourced from a school cafeteria and reporting usually consuming 5 school lunches per week, or
3). Consuming 2 school lunch components sourced from a school cafeteria and reporting zero food sourced outside of a school cafeteria during the meal.

### Nutrient intake

The first 24-h dietary recall was utilized to determine total daily nutrient intake among school lunch consumers and nonconsumers, nutrient intake only from school lunches, and nutrient intake from beef-containing school lunch meals. Usual nutrient intake, which was used for estimating the percentage of the population < EAR or > AI, was calculated using both 24-h dietary recalls and the National Cancer Institute method [19].

### Statistical analyses

SAS 9.4 (SAS Institute) was used for all analyses. All analyses were adjusted for the complex sampling procedure used in NHANES with appropriate primary sampling units, strata, and dietary day 1 survey weights. Regression analyses in combination with a t-test were used to determine whether significant differences in nutrient intake were present between beef consumers compared with nonconsumers at school lunch. Analyses were adjusted for age, sex, race/ethnicity, and energy intake. Significant differences in the percentage below the EAR/above the AI were determined with a *z*-test. Significance for all analyses was set at *P* < 0.05.

## Results

### Sample characteristics

The analytical sample included 3046 individuals (weighted *n* = 18,787,280) who consumed school lunch. Of these, 1153 individuals (weighted *n* = 7,024,904) consumed beef at school lunch, and 1893 individuals (weighted *n* = 11,762,376) did not consume beef at school lunch. A higher percentage of consumers of beef at school lunch were Mexican American compared with nonconsumers of beef at school lunch; no other differences were observed (Table 1). Overall, beef consumers at school lunch had a median beef intake of 14 g [interquartile range (IQR): 5–41; Supplementary Table 1].

**TABLE 1:** Characteristics of the total sample, school lunch consumers and nonconsumers, and consumers and nonconsumers of beef at school lunch

Variables	Total	School lunch		School Lunch Consumers	
		Consumers	Nonconsumers	Beef consumed	Beef not consumed
Sample *n*	19,482	3046	16,436	1153	1893
Weighted *n*	114,757,656	18,787,280	95,970,376	7,024,904	11,762,376
Gender					
Male	50.83 ± 0.62	53.55 ± 1.45^1^	50.30 ± 0.64	56.82 ± 1.98	51.59 ± 1.99
Age, mean ± SE	11.59 ± 0.06	10.77 ± 0.11^1^	11.75 ± 0.06	10.72 ± 0.17	10.79 ± 0.15
Age Categories					
5–8 y	27.96 ± 0.58	33.06 ± 1.29^1^	26.97 ± 0.65	34.13 ± 2.18	32.42 ± 1.61
9–13 y	35.13 ± 0.51	40.03 ± 1.17^1^	34.17 ± 0.61	38.11 ± 1.94	41.18 ± 1.49
14–18 y	36.90 ± 0.66	26.91 ± 1.42^1^	38.86 ± 0.74	27.77 ± 2.42	26.40 ± 1.65
BMI					
Underweight	3.44 ± 0.22	3.05 ± 0.46	3.52 ± 0.26	3.57 ± 0.71	2.73 ± 0.65
Normal weight	61.79 ± 0.67	59.41 ± 1.41	62.26 ± 0.73	59.06 ± 2.29	59.62 ± 1.79
Overweight	15.95 ± 0.41	15.58 ± 0.96	16.02 ± 0.45	14.94 ± 1.51	15.96 ± 1.28
Obesity	18.81 ± 0.56	21.97 ± 1.23^1^	18.20 ± 0.56	22.43 ± 2.34	21.69 ± 1.21
Race and Ethnicity					
Non-Hispanic White	56.72 ± 1.57	47.23 ± 2.84^1^	58.58 ± 1.53	44.53 ± 3.48	48.84 ± 2.95
Non-Hispanic Black	14.09 ± 0.87	19.15 ± 1.56^1^	13.10 ± 0.83	19.35 ± 1.82	19.03 ± 1.69
Mexican American	14.41 ± 0.99	18.49 ± 1.85^1^	13.62 ± 0.91	21.67 ± 2.48^2^	16.59 ± 1.71
Other Hispanic	6.45 ± 0.52	6.94 ± 0.82	6.35 ± 0.55	6.39 ± 1.03	7.27 ± 1.05
Other race, including multiracial	8.33 ± 0.47	8.20 ± 0.92	8.35 ± 0.50	8.07 ± 1.42	8.27 ± 1.05
Food Security Status					
Food secure	88.61 ± 0.51	86.21 ± 1.21^1^	89.10 ± 0.50	84.45 ± 1.99	87.28 ± 1.28
Low food security	9.77 ± 0.44	11.35 ± 1.07	9.45 ± 0.44	13.85 ± 1.95	9.85 ± 1.05
Very low food security	1.61 ± 0.20	2.43 ± 0.55	1.45 ± 0.18	1.71 ± 0.50	2.87 ± 0.80
Poverty to Income Ratio					
<1.35	33.00 ± 1.08	42.08 ± 2.25^1^	31.19 ± 1.04	44.35 ± 2.88	40.72 ± 2.56
1.35–1.85	11.05 ± 0.49	14.44 ± 1.16^1^	10.37 ± 0.50	13.27 ± 2.03	15.15 ± 1.28
>1.85	55.95 ± 1.20	43.47 ± 2.32^1^	58.44 ± 1.18	42.39 ± 2.90	44.13 ± 2.70

Least squares mean % ± SEM unless otherwise stated.

1 *P* < 0.05 compared with school lunch nonconsumers.

2 *P* < 0.05 compared with nonconsumers of beef at school lunch.

### Total daily nutrient intake in school lunch consumers compared with nonconsumers

School lunch consumers had higher intake of calcium (146 ± 19 mg; *P* < 0.001), fiber (1.1 ± 0.2 g; *P* < 0.001), phosphorus (117 ± 19 mg; *P* < 0.001), potassium (207 ± 35 mg; *P* < 0.001), protein (3.7 ± 1.1 g; *P* = 0.001), magnesium (8.9 ± 3.4 mg; *P* = 0.01), riboflavin (0.2 ± 0.03 mg, *P* < 0.001), thiamin (0.06 ± 0.03 mg; *P* = 0.02), and vitamins A (74 ± 17 RE; *P* < 0.001), B12 (0.4 ± 0.1 μg; *P* = 0.003), and D (1.3 ± 0.2 μg; *P* < 0.001), and zinc (0.6 ± 0.2 mg; *P* = 0.003), and lower intake of vitamin E (−0.4 ± 0.1 mg; *P* =0.01) compared with nonconsumers of school lunch (Table 2). For school lunch consumers, school lunch provided ∼17% of total daily energy intake. For consumers of beef at school lunch, beef provided ∼39% of energy consumed at school lunch, which equates to ∼6% of total daily energy intake (Table 2). The most commonly consumed greatest contributors to beef intake in school lunch consumers were: meat mixed dishes (*n* consumers = 105; mean intake: 59 ± 4.5 g), burritos and tacos (*n* = 105; 51 ± 2.2 g), burgers (*n =* 54; 42 ± 1.0 g), Frankfurter sandwiches (*n =* 144; 39 ± 4.1 g), nachos (*n =* 64; 39 ± 1.5 g), pasta mixed dishes excluding macaroni and cheese (*n =* 89; 31 ± 1.2 g), and pizza (*n =* 564; 5.6 ± 0.6 g) (Supplementary Table 2).

**TABLE 2:** Total daily nutrient intake for school lunch consumers and nonconsumers and contribution of school lunch to daily intake for children 5 to 18 y

	Total daily nutrient intake		School lunch intake for NSLP consumers	
	mean ± SEM		mean ± SEM (% of daily intake ± SEM) [% contribution of beef to nutrient intake from school lunch]	
	NSLP consumers	NSLP nonconsumers	All foods	Beef only
Energy, kcal	2049 ± 21	2026 ± 11	339 ± 7.3 (16.5 ± 0.3%)	131 ± 7.0 (6.4 ± 0.3%) [38.6%]
Protein, g	74.9 ± 1.0^1^	71.2 ± 0.5	15.9 ± 0.4 (21.3 ± 0.5%)	6.4 ± 0.3 (8.5 ± 0.4%) [40.3%]
Total fat, g	76.5 ± 1.0	76.6 ± 0.5	15.7 ± 0.4 (20.6 ± 0.4%)	6.2 ± 0.3 (8.1 ± 0.4%) [39.5%]
Saturated fatty acids, g	27.2 ± 0.4	26.6 ± 0.2	5.3 ± 0.1 (19.6 ± 0.5%)	2.3 ± 0.1 (8.5 ± 0.4%) [43.4%]
Carbohydrates, g	271 ± 2.8	268 ± 1.4	33.4 ± 0.8 (12.3 ± 0.3%)	12.5 ± 0.7 (4.6 ± 0.3%) [37.4%]
Dietary fiber, g	15.0 ± 0.2^1^	13.9 ± 0.1	2.6 ± 0.1 (17.3 ± 0.5%)	1.0 ± 0.1 (6.8 ± 0.4%) [38.5%]
Calcium, mg	1146 ± 16^1^	1001 ± 9	159 ± 5.6 (13.9 ± 0.5%)	65.0 ± 3.9 (5.7 ± 0.3%) [40.9%]
Choline, mg	259 ± 5	258 ± 2	39.0 ± 1.3 (15.1 ± 0.5%)	15.8 ± 1.0 (6.1 ± 0.4%) [40.5%]
Copper, mg	1.0 ± 0.02	1.0 ± 0.01	0.2 ± 0.01 (14.8 ± 0.5%)	0.1 ± 0.004 (6.0 ± 0.4%) [50.0%]
Folate, DFE μg	545 ± 9.1	538 ± 5.2	84.9 ± 3.0 (15.6 ± 0.6%)	37.7 ± 2.4 (6.9 ± 0.4%) [44.4%]
Iron, mg	14.8 ± 0.2	14.7 ± 0.1	2.4 ± 0.1 (16.1 ± 0.4%)	1.1 ± 0.1 (7.3 ± 0.4%) [45.8%]
Magnesium, mg	247 ± 3.1^1^	238 ± 1.6	36.4 ± 1.0 (14.7 ± 0.4%)	13.6 ± 0.8 (5.5 ± 0.3%) [37.4%]
Niacin, mg	22.5 ± 0.3	22.5 ± 0.2	4.4 ± 0.1 (19.7 ± 0.4%)	1.6 ± 0.1 (7.0 ± 0.4%) [36.4%]
Phosphorus, mg	1394 ± 17^1^	1277 ± 9	253 ± 6.3 (18.1 ± 0.4%)	95.4 ± 5.0 (6.8 ± 0.3) [37.7%]
Potassium, mg	2391 ± 30^1^	2184 ± 17	312 ± 9.1 (13.0 ± 0.4%)	124 ± 8.1 (5.2 ± 0.3) [39.7%]
Riboflavin, mg	2.2 ± 0.03^1^	2.0 ± 0.02	0.3 ± 0.01 (12.4 ± 0.4%)	0.1 ± 0.01 (5.1 ± 0.3) [33.3%]
Selenium, μg	101 ± 1	99 ± 1	23.8 ± 0.7 (23.5 ± 0.5%)	9.6 ± 0.5 (9.5 ± 0.5) [40.3%]
Sodium, mg	3275 ± 43	3235 ± 24	698 ± 18.0 (21.3 ± 0.5%)	290 ± 14.3 (8.8 ± 0.4) [41.5%]
Thiamin, mg	1.7 ± 0.02^1^	1.6 ± 0.01	0.3 ± 0.01 (16.8 ± 0.6%)	0.1 ± 0.01 (7.1 ± 0.4) [33.3%]
Vitamin A, retinol equivalents	656 ± 15^1^	581 ± 6.8	61.1 ± 2.3 (9.3 ± 0.4%)	23.1 ± 1.9 (3.5 ± 0.3%) [37.8%]
Vitamin B6, mg	1.8 ± 0.03	1.8 ± 0.02	0.2 ± 0.01 (13.0 ± 0.5%)	0.1 ± 0.01 (4.5 ± 0.3%) [50.0%]
Vitamin B12, μg	5.3 ± 0.1^1^	4.9 ± 0.1	0.7 ± 0.03 (14.1 ± 0.5%)	0.4 ± 0.02 (7.6 ± 0.4%) [57.1%]
Vitamin C, mg	81.1 ± 3.5	76.0 ± 1.2	2.5 ± 0.2 (3.1 ± 0.3%)	0.9 ± 0.1 (1.1 ± 0.2%) [36.0%]
Vitamin D (D2 + D3), μg	6.5 ± 0.1^1^	5.2 ± 0.07	0.3 ± 0.02 (4.2 ± 0.3%)	0.1 ± 0.01 (1.2 ± 0.1%) [33.3%]
Vitamin E, mg	6.6 ± 0.1^1^	7.0 ± 0.1	1.2 ± 0.03 (18.0 ± 0.4%)	0.4 ± 0.02 (5.9 ± 0.4%) [8.3%]
Zinc, mg	11.2 ± 0.2^1^	10.6 ± 0.1	1.9 ± 0.06 (17.1 ± 0.5%)	1.0 ± 0.1 (8.9 ± 0.5%) [52.6%]

Abbreviation: DFE, dietary folate equivalents.

1 *P* < 0.05 compared with NSLP nonconsumers.

### Total daily nutrient intake in consumers of beef at school lunch compared with nonconsumers of beef at school lunch

Consumers of beef at school lunch had higher intake of saturated fat (2.3 ± 0.5 g; *P* < 0.001), total fat (2.0 ± 1.0 g; *P* = 0.04), vitamin B12 (0.6 ± 0.2 μg; *P* = 0.003), and zinc (1.1 ± 0.3 mg; *P*< 0.001) compared with nonconsumers of beef at school lunch (Table 3). Furthermore, intake of carbohydrates (−6.2 ± 2.8 g; *P* = 0.03), niacin (−1.4 ± 0.4 mg; *P* = 0.002), and vitamin E (−0.5 ± 0.2 mg; *P* < 0.001) were lower in consumers of beef at school lunch compared with nonconsumers of beef at school lunch. When examining food group intake, consumers of beef at school lunch had higher total daily intake of meat (0.67 ± 0.13 oz-eq; *P* < 0.001) and cured meat (0.30 ± 0.08 oz-eq; *P* < 0.001), and lower intake of nuts and seeds (−0.12 ± 0.04 oz-eq; *P* = 0.006), poultry (−1.05 ± 0.10 oz-eq; *P* < 0.001), and seafood high in omega (ω)-3 (−0.02 ± 0.01 oz-eq; *P* = 0.03) compared with nonconsumers of beef at school lunch (Supplementary Table 3). Added sugar intake tended to be lower in beef consumers at school lunch compared with beef nonconsumers at school lunch (−1.07 ± 0.56 tsp-eq; *P* = 0.06).

**TABLE 3:** Total daily nutrient intake of consumers of beef at school lunch, compared with nonconsumers of beef at school lunch, overall, in children aged 5 to 18 y

Nutrient	Beef consumer *n* = 1153	Beef nonconsumer *n* = 1893	Difference
Energy, kcal	2068 ± 37	2035 ± 24	33 ± 45
Protein, g	75.6 ± 1.0	74.6 ± 0.7	1.0 ± 1.2
Total fat, g	77.9 ± 0.8	75.8 ± 0.6	2.0 ± 1.0∗
Saturated fat, g	28.6 ± 0.4	26.4 ± 0.3	2.3 ± 0.5∗
Carbohydrate, g	267 ± 2.2	274 ± 1.7	−6.2 ± 2.8∗
Dietary fiber, g	15.0 ± 0.2	15.0 ± 0.2	0.0 ± 0.3
Calcium, mg	1176 ± 18.6	1133 ± 17.5	42.6 ± 26.5
Sodium, mg	3313 ± 30.3	3257 ± 34.5	55.4 ± 38.9
Potassium, mg	2425 ± 28.9	2378 ± 27.2	46.9 ± 33.7
Copper, mg	1.04 ± 0.01	1.05 ± 0.02	−0.02 ± 0.02
Folate, DFE μg	552 ± 13.1	543 ± 11.4	9.0 ± 17.5
Iron, mg	14.9 ± 0.2	14.7 ± 0.2	0.2 ± 0.3
Magnesium, mg	243 ± 2.7	250 ± 2.6	−6.8 ± 3.5
Niacin, mg	21.6 ± 0.3	23.0 ± 0.3	−1.4 ± 0.4∗
Phosphorus, mg	1403 ± 15.4	1393 ± 12.7	10.3 ± 18.9
Selenium, μg	102 ± 1.5	101 ± 1.1	1.4 ± 1.7
Vitamin A, retinol equivalents	660 ± 18.2	656 ± 17.8	3.3 ± 23.2
Thiamin, mg	1.7 ± 0.03	1.7 ± 0.02	0.03 ± 0.03
Riboflavin, mg	2.2 ± 0.03	2.2 ± 0.03	0.03 ± 0.04
Vitamin B6, mg	1.7 ± 0.04	1.8 ± 0.03	−0.1 ± 0.05
Vitamin B12, μg	5.7 ± 0.1	5.1 ± 0.1	0.6 ± 0.2∗
Vitamin C, mg	79.1 ± 3.6	82.5 ± 4.7	−3.3 ± 5.9
Vitamin D (D2 + D3), μg	6.6 ± 0.2	6.5 ± 0.2	0.1 ± 0.2
Vitamin E (α tocopherol), mg	6.3 ± 0.1	6.9 ± 0.1	−0.5 ± 0.2∗
Zinc, mg	11.9 ± 0.2	10.8 ± 0.2	1.1 ± 0.3∗
Choline, mg	264 ± 4.8^1^	255 ± 4.3^2^	8.8 ± 5.6

Data presented as least squares mean ± SE.

∗ *P* < 0.05.

1 *n* = 1002.

2 *n* = 1657.

The nutrient intake of consumers of beef at school lunch, compared with nonconsumers of beef at school lunch, by gender and age category, is shown in Supplementary Table 4. In males aged 5 to 8 y, sodium intake was higher (186 ± 81 mg; *P* = 0.02) and choline intake was lower (−25 ± 11.6 mg; *P* = 0.03) in beef consumers at school lunch compared with beef nonconsumers at school lunch. In males and females 9 to 13 years, intake of saturated fat (males 3.1 ± 1.0 g, *P* = 0.002; females 2.1 ± 0.9 g, *P* = 0.02), vitamin B12 (males 1.0 ± 0.5 μg, *P* = 0.03; females 0.6 ± 0.3 μg, *P* = 0.02), and zinc (males 1.9 ± 0.7 mg, *P* = 0.009; females 1.2 ± 0.4 mg, *P* = 0.004) were higher in beef consumers at school lunch compared with beef nonconsumers. In males 9 to 13 y only, energy (248 ± 119 kcal; *P* = 0.04) and choline (33.4 ± 15.2 mg; *P* = 0.03) intake were higher in beef consumers compared with nonconsumers at school lunch, and in females only niacin (−1.6 ± 0.8 mg; *P* = 0.04) intake was lower in beef consumers compared with nonconsumers at school lunch. For males and females 14 to 18 y, saturated fat (males 5.4 ± 1.7 g, *P* = 0.002; females 3.8 ± 1.0 g, *P* < 0.001) intake was higher in beef consumers compared with nonconsumers at school lunch. For males 14 to 18 y only, intake of total fat (7.0 ± 3.0 g; *P* = 0.02), calcium (226 ± 90 mg; *P* = 0.01), phosphorus (181 ± 59 mg; *P* = 0.003), vitamin B12 (1.3 ± 0.5 μg; *P* = 0.01) and zinc (2.0 ± 0.9 mg; *P* = 0.03) was higher in beef consumers compared with nonconsumers at school lunch, and carbohydrate intake was lower (−25.8 ± 9.0 g; *P* = 0.005). In females 14 to 18 y only, intake of fiber (−1.8 ± 0.8 g; *P* = 0.03), copper (−0.1 ± 0.03 mg; *P* = 0.02), magnesium (−29 ± 8.2 mg; *P* < 0.001), niacin (−4.5 ± 1.1 mg; *P* < 0.001), selenium (−9.0 ± 3.2; *P* = 0.007), vitamin B6 (−0.3 ± 0.1 mg; *P* = 0.03), and vitamin E (−0.8 ± 0.4 mg; *P* = 0.03) were lower in beef consumers compared with nonconsumers at school lunch.

The nutrient intake of consumers of beef at school lunch compared with nonconsumers of beef at school lunch by food security status category is shown in Supplementary Table 5. In food secure individuals, beef intake at school lunch was associated with higher intake of total fat (2.6 ± 1.1 g; *P* = 0.02), saturated fat (2.6 ± 0.6 g; *P* < 0.001), choline (12.6 ± 5.9 mg; *P* = 0.04), vitamin B12 (0.7 ± 0.2 μg; *P* = 0.002) and zinc (1.2 ± 0.3 mg; *P* < 0.001) compared with nonconsumers of beef at school lunch. In addition, intake of carbohydrates (−7.7 ± 3.3 g; *P* = 0.02), niacin (−1.2 ± 0.5 mg; *P* = 0.008), and vitamin E (−0.5 ± 0.2 mg; *P* = 0.006) were lower in beef consumers at school lunch compared with nonconsumers of beef at school lunch. In individuals with low food security, beef intake at school lunch was associated with lower intake of magnesium (−18.7 ± 8.5 mg; *P* = 0.03), vitamin A (−123 ± 50 RE; *P* = 0.02), and vitamin E (−0.7 ± 0.4 mg; *P* = 0.04) compared with nonconsumers of beef at school lunch. In those with very low food security, beef consumers at school lunch had higher intake of zinc (3.8 ± 1.7 mg; *P* = 0.03) and lower intake of vitamin A (−223 ± 62 RE; *P* < 0.001) and vitamin C (−28 ± 11 mg; *P* = 0.01) compared with those who did not consume beef at school lunch.

### Nutrient adequacy in consumers of beef at school lunch compared with nonconsumers of beef at school lunch

A lower percentage of consumers of beef at school lunch, compared with nonconsumers of beef at school lunch, had a zinc intake (−3.94 ± 1.97 percentage points; *P* = 0.045) less than the EAR (Table 4). When comparing the nutrient adequacy of consumers of beef at school lunch with nonconsumers of beef at school lunch across age and gender categories, limited differences were observed (Supplementary Table 6). For females in the 5 to 8 and 9 to 13 y age categories, a higher percentage of beef consumers were below the EAR for vitamin E (5 to 8 y: 15.50 ± 7.50 percentage points, *P* = 0.04; 9 to 13 y: 8.11 ± 3.53 percentage points, *P* = 0.02) compared with nonconsumers of beef at school lunch. For males in the 9 to 13 y age group, a higher percentage of beef consumers were above the AI for potassium (13.96 ± 6.72 percentage points; *P* = 0.04) compared with nonconsumers of beef at school lunch.

**TABLE 4:** The percentage of consumers of beef at school lunch, compared with nonconsumers of beef at school lunch, who have a nutrient intake below the estimated average requirement (EAR)/above the adequate intake (AI) in children aged 5 to 18 y

Nutrient	Beef consumer *n* = 1153	Beef nonconsumer *n* = 1893	Difference
Calcium	33.23 ± 3.02	39.60 ± 2.07	−6.37 ± 3.66
Choline^1^	21.53 ± 2.47^2^	17.43 ± 1.41^3^	4.11 ± 2.84
Copper	1.49 ± 0.71	2.06 ± 0.68	−0.57 ± 0.98
Folate	4.09 ± 1.39	2.91 ± 0.85	1.18 ± 1.62
Iron	1.62 ± 0.41	1.76 ± 0.44	−0.14 ± 0.60
Magnesium	32.80 ± 2.60	32.05 ± 1.75	0.75 ± 3.13
Niacin	0.27 ± 0.18	0.09 ± 0.09	0.18 ± 0.20
Phosphorus	8.72 ± 1.67	9.42 ± 1.33	−0.70 ± 2.13
Potassium^1^	47.18 ± 2.91	41.87 ± 2.13	5.30 ± 3.61
Riboflavin	0.41 ± 0.24	0.40 ± 0.20	0.02 ± 0.32
Selenium	0.01 ± 0.03	0.02 ± 0.02	0.00 ± 0.03
Sodium^1^	100 ± 0.00	99.97 ± 0.02	0.02 ± 0.02
Thiamin	0.89 ± 0.55	0.72 ± 0.29	0.16 ± 0.62
Vitamin A	20.46 ± 2.87	21.37 ± 2.48	−0.92 ± 3.79
Vitamin B6	2.77 ± 1.23	1.62 ± 0.67	1.15 ± 1.40
Vitamin B12	0.10 ± 0.12	0.76 ± 0.36	−0.66 ± 0.38
Vitamin C	18.10 ± 2.81	18.02 ± 3.40	0.08 ± 4.41
Vitamin D (D2 + D3)	87.01 ± 2.26	88.32 ± 1.45	−1.31 ± 2.69
Vitamin E (α tocopherol)	78.99 ± 2.36	73.71 ± 1.75	5.27 ± 2.93
Zinc	2.89 ± 1.16	6.83 ± 1.59	−3.94 ± 1.97∗

Data are the percentage below the EAR ± SEM unless otherwise stated.

1 above the adequate intake level.

2 *n* = 1002.

3 *n* = 1657.

∗ *P* < 0.05.

When examining the nutrient adequacy of consumers of beef at school lunch compared with nonconsumers of beef at school lunch across food security status categories, several differences were observed. For food-secure individuals, a lower percentage of beef consumers had a calcium (−7.14 ± 3.58 percentage points; *P* = 0.046) intake below the EAR compared with nonconsumers of beef at school lunch. For individuals with low food security, a higher percentage of beef consumers had a vitamin D (14.83 ± 6.91 percentage points; *P* = 0.03) intake below the EAR compared to nonconsumers of beef at school lunch. For individuals with very low food security, a lower percentage of beef consumers at school lunch had iron (−7.76 ± 2.23 percentage points; *P* < 0.001), magnesium (−23.40 ± 11.62 percentage points; *P* = 0.04) and vitamin B12 (−5.09 ± 2.43 percentage points; *P* = 0.04) intake below the EAR compared with nonconsumers of beef at school lunch (Table 5).

**TABLE 5:** The percentage of consumers of beef at school lunch, compared with nonconsumers of beef at school lunch, who have a nutrient intake below the estimated average requirement (EAR)/above the adequate intake (AI) level by food security category

Nutrient	Food secure			Low food security			Very low food security		
	Beef consumer *n* = 939	Beef nonconsumer *n* = 1578	Difference	Beef consumer *n* = 171	Beef nonconsumer *n* = 223	Difference	Beef consumer *n* = 19	Beef nonconsumer *n* = 51	Difference
Calcium	31.18 ± 2.78	38.32 ± 2.25	−7.14 ± 3.58∗	46.35 ± 7.58	40.13 ± 6.94	6.22 ± 10.27	19.41 ± 27.68	55.43 ± 10.78	−36.02 ± 29.70
Choline^1^	23.30 ± 2.70^2^	17.27 ± 1.48^3^	6.03 ± 3.08	16.58 ± 7.42^4^	18.57 ± 3.64^5^	−1.98 ± 8.27	22.83 ± 3.88^6^	13.01 ± 6.75^7^	9.82 ± 7.78
Copper	1.24 ± 0.84	1.60 ± 0.63	−0.36 ± 1.05	3.53 ± 3.51	5.98 ± 2.06	−2.44 ± 4.07	0.00 ± 0.00	2.49 ± 4.47	−2.49 ± 4.47
Folate	3.26 ± 1.23	2.53 ± 0.81	0.73 ± 1.47	8.36 ± 6.94	5.60 ± 2.24	2.76 ± 7.29	4.11 ± 5.28	16.70 ± 6.71	−12.58 ± 8.54
Iron	1.28 ± 0.43	1.51 ± 0.43	−0.23 ± 0.61	3.54 ± 2.15	4.07 ± 1.53	−0.53 ± 2.64	0.00 ± 0.28	7.76 ± 2.21	−7.76 ± 2.23∗
Magnesium	30.97 ± 2.69	30.36 ± 1.93	0.61 ± 3.31	40.41 ± 7.27	29.94 ± 5.55	10.46 ± 9.15	36.52 ± 3.21	59.93 ± 11.16	−23.40 ± 11.62∗
Niacin	0.21 ± 0.16	0.04 ± 0.09	0.17 ± 0.18	0.69 ± 2.50	0.51 ± 0.57	0.18 ± 2.56	0.00 ± 0.40	0.39 ± 3.03	−0.39 ± 3.06
Phosphorus	8.64 ± 1.67	8.69 ± 1.39	−0.05 ± 2.17	7.83 ± 8.39	12.83 ± 3.47	−5.00 ± 9.08	1.69 ± 15.63	18.94 ± 22.09	−17.25 ± 27.05
Potassium^1^	46.91 ± 3.26	41.88 ± 2.16	5.04 ± 3.91	44.55 ± 8.45	50.80 ± 6.13	−6.25 ± 10.44	91.03 ± 50.39	7.44 ± 16.56	83.59 ± 53.04
Riboflavin	0.41 ± 0.23	0.31 ± 0.17	0.10 ± 0.29	0.59 ± 1.85	1.93 ± 1.01	−1.34 ± 2.11	0.23 ± 1.18	0.26 ± 0.58	−0.03 ± 1.32
Selenium	0.01 ± 0.02	0.01 ± 0.02	0.003 ± 0.03	0.02 ± 0.51	0.14 ± 0.25	−0.12 ± 0.57	0.00 ± 0.02	0.00 ± 0.00	0.00 ± 0.02
Sodium^1^	100 ± 0.01	99.98 ± 0.02	0.02 ± 0.02	100 ± 0.08	99.74 ± 0.24	0.26 ± 0.25	100 ± 0.03	100 ± 0.00	0.00 ± 0.03
Thiamin	0.74 ± 0.43	0.46 ± 0.25	0.28 ± 0.50	3.15 ± 4.37	2.27 ± 1.23	0.87 ± 4.54	0.24 ± 0.66	1.06 ± 2.91	−0.82 ± 2.99
Vitamin A	19.06 ± 2.88	21.01 ± 2.86	−1.95 ± 4.06	31.19 ± 8.10	20.59 ± 5.55	10.59 ± 9.82	0.00 ± 18.91	22.15 ± 21.01	−22.15 ± 28.27
Vitamin B6	2.17 ± 0.93	1.53 ± 0.66	0.65 ± 1.14	9.48 ± 9.42	1.71 ± 1.54	7.77 ± 9.55	0.00 ± 0.57	3.56 ± 5.84	−3.56 ± 5.86
Vitamin B12	0.13 ± 0.12	0.64 ± 0.37	−0.50 ± 0.38	0.10 ± 0.55	0.91 ± 0.81	−0.81 ± 0.98	0.07 ± 0.30	5.17 ± 2.41	−5.09 ± 2.43∗
Vitamin C	16.57 ± 2.86	18.43 ± 3.62	−1.87 ± 4.61	24.59 ± 10.54	10.98 ± 5.68	13.61 ± 11.97	11.10 ± 27.64	30.11 ± 20.00	−19.00 ± 34.12
Vitamin D (D2 + D3)	85.64 ± 2.49	88.61 ± 1.38	−2.97 ± 2.85	97.93 ± 1.86	83.11 ± 6.65	14.83 ± 6.91∗	71.80 ± 10.38	91.13 ± 4.42	−19.33 ± 11.28
Vitamin E (α tocopherol)	77.21 ± 2.71	73.60 ± 2.01	3.61 ± 3.37	86.04 ± 9.95	69.21 ± 3.88	16.82 ± 10.68	81.80 ± 7.01	74.79 ± 10.81	7.01 ± 12.88
Zinc	2.85 ± 1.27	5.85 ± 1.66	−2.99 ± 2.09	4.06 ± 3.36	12.46 ± 3.88	−8.40 ± 5.14	0.00 ± 0.00	19.18 ± 10.44	−19.18 ± 10.44

Data are the percentage below the EAR ± SEM unless otherwise stated.

1 above the adequate intake level; presented n are unweighted.

2 *n* = 821.

3 *n =* 1395.

4 *n* = 146.

5 *n* = 184.

6 n = 15.

7 *n* = 48.

∗ *P* < 0.05.

## Discussion

These analyses of NHANES 2003−2018 assessed the contribution of beef consumed as part of the NSLP to nutrient intake and nutrient adequacy. Overall, beef intake at school lunch was low. However, beef consumers at school lunch had higher intakes of total fat and saturated fat, vitamin B12 and zinc, and lower intakes of carbohydrates, niacin, and vitamin E compared with beef nonconsumers at school lunch. Furthermore, a lower percentage of beef consumers at school lunch had a zinc intake less than the EAR compared with beef nonconsumers at school lunch. Some differences in nutrient intake and adequacy with beef intake at school lunch were also observed by gender and age category, as well as food security status. For those with very low food security, a lower percentage of beef consumers at school lunch had iron, magnesium, and vitamin B12 intake below the EAR compared with nonconsumers of beef at school lunch. Collectively, these analyses show relatively modest and limited differences in nutrient intake and adequacy among beef consumers and nonbeef consumers at school lunch, which is likely because of the observed low beef intake (median 14 g) as well as the NSLP nutrition standards that ensure provision of nutritious meals.

Previously, it was reported that beef significantly contributes to micronutrient intake in children and adolescents [13,14], and in adolescents, beef intake is associated with greater nutrient adequacy [14]. In an analysis of NHANES from 1999 to 2004, beef intake contributed significantly to intake of iron, niacin, phosphorus, and potassium, vitamins B6 and B12, and zinc [13]. Thus, our finding that beef consumers at school lunch had higher intakes of vitamin B12 and zinc is aligned with these prior findings, although we observed lower intake of niacin and did not see any differences in the intake of the other nutrients. In our study, beef consumers at school lunch had intakes of vitamin B12 and zinc that were ∼10% higher than nonconsumers of beef. For zinc, this translated into fewer beef consumers at school lunch being below the EAR (∼4 percentage points) than nonbeef consumers. Collectively, these findings suggest that beef intake as part of the NSLP has a relatively modest impact on overall nutrient intake, likely because of low beef consumption. Higher beef intake at school lunch would likely be needed to influence micronutrient intake and adequacy.

In a previous analysis of NHANES from 2001 to 2018, a lower percentage of adolescents (age 14 to 18 y) who consumed beef, compared with those who did not consume beef, had intakes less than the EAR for several micronutrients [14]. In addition, male and female adolescents who consumed beef had higher intakes of calcium, iron, niacin, phosphorus, potassium, selenium, sodium, thiamin, choline, vitamin B12, and zinc compared with beef nonconsumers [14]. In our analyses, no differences in nutrient adequacy were observed between adolescents who consumed beef as part of the NSLP compared with those who did not. However, we observed that female adolescents who consumed beef at school lunch had lower intakes of fiber, copper, magnesium, niacin, vitamin B6, vitamin E, and selenium compared with nonconsumers; male beef consumers at school lunch had higher intakes of calcium, phosphorus, vitamin B12, and zinc compared with nonconsumers. Although our findings for male adolescents are somewhat aligned with Fulgoni et al. [14] , our results for female adolescents differ. The reason(s) for these discrepant findings is unclear. The low intake of beef at NSLP by adolescents may be a contributing factor that limited our ability to detect the contribution of beef at school lunch to nutrient intake/adequacy. In adolescents who participated in the NSLP and consumed beef, the median beef intake was 8 g (8 g in males; 6 g in females), which is ∼17% of their total daily beef intake (47 g). Given this low beef intake, it is unlikely that the observed differences in nutrient intakes in adolescents who consumed beef at school lunch were caused by beef intake per se. These findings should be interpreted cautiously, given risk of confounding.

In the overall analyses, saturated fat intake was higher in beef consumers at school lunch, compared with beef nonconsumers by ∼3%; higher saturated fat intake was also seen across several of the analyses by gender and age category, as well as in those classified as food secure. Based on the food group analyses, it appears that beef was displacing poultry, nuts and seeds, and seafood (high ω-3) in beef consumers at school lunch. These foods are typically lower in saturated fat, which may explain these findings. In addition, processed (cured) meat intake was higher in beef consumers compared with nonconsumers at school lunch, which may also have contributed to the higher saturated fat intake because processed meats tend to be higher in saturated fat. Given these findings, it is pertinent to continue to emphasize the inclusion of lean meat in the NSLP as well as other meats/meat alternatives that are low in saturated fat and limit processed meats. This is because saturated fat is overconsumed in the United States and, therefore, is designated as a nutrient of public health concern for overconsumption because it is related to higher risk of cardiovascular disease [20].

Given prior research showing food insecurity is associated with a higher risk of nutrient inadequacy in US children [15], we conducted analyses by food insecurity status. We found that for individuals with very low food security, a lower percentage of beef consumers at school lunch had iron, magnesium, and vitamin B12 intake less than the EAR compared with nonconsumers of beef at school lunch. Jun et al. [15] found that boys and girls with food insecurity had a higher prevalence of inadequate intake of magnesium than their food-secure counterparts. Our findings suggest that intake of beef as part of the NSLP may assist with ensuring adequate consumption of iron, magnesium, and vitamin B12 in those with very low food security who are vulnerable to micronutrient inadequacy.

Previous research has shown that participation in the NSLP is associated with higher diet quality compared with nonparticipation [2,3]. To our knowledge, micronutrient intake between school lunch consumers and nonconsumers has not been previously examined. Our analyses show that NSLP consumers had higher intake of protein, fiber, calcium, magnesium, phosphorus, potassium, riboflavin, thiamin, vitamin B12 and D, and zinc compared with NSLP nonconsumers. These findings further demonstrate the contribution that the NSLP makes to ensuring dietary adequacy in children and adolescents.

These analyses represent the first investigation of the contribution that beef consumed as part of the NSLP has on nutrient intake and nutrient adequacy. Strengths of the analyses are the use of a nationally representative dataset and use of a previously established method to determine NSLP participation. However, the findings of these analyses should be interpreted considering some limitations. First, although an established method was used to determine NSLP participation, this approach has not been validated, and therefore, misclassification may have occurred. Second, reported energy intake of NSLP participants was 16.5% of total daily energy. An analysis of NHANES 2017 to March 2020 data showed lunch from all sources, including school and home, comprised 29% of total energy for individuals 2 to 19 y [21]. Although this discrepancy could be because of energy differences between lunch sources, it is also possible that intake underreporting may have occurred. Finally, a relatively small number of individuals were consumers of beef at school lunch, which may have limited our statistical power. This is particularly an issue for the food security analysis.

We used NHANES 2003–2018 to examine nutrient intake and nutrient adequacy in students consuming beef as part of the NSLP, compared with students consuming an NSLP meal without beef, overall, by age and gender category, and by food security status. Overall, beef intake as part of the NSLP was associated with ∼10% higher intake of zinc and vitamin B12, ∼4% higher intake of saturated fat, as well as lower intake of niacin (−6%) and vitamin E (−7%). In addition, fewer beef consumers at school lunch were below the EAR for zinc (∼4 percentage points) compared with nonbeef consumers at school lunch. These results suggest beef intake as part of the NSLP has a relatively modest and limited contribution to overall nutrient intake, likely because the NSLP nutrition standards mandate the provision of nutritious meals, and overall beef consumption was low.

## Author contributions

The authors’ responsibilities were as follows – KSP, KF, and VLF designed the research; KF and VLF conducted the data analyses; KSP, KF, and VLF interpreted the data; KSP drafted the manuscript; KF and VLF critically reviewed the manuscript; and all authors have read and approved the final manuscript.

## Data availability

The data used in this study are openly available in the NHANES website: NHANES Questionnaires, Datasets, and Related Documentation (https://wwwn.cdc.gov/nchs/nhanes/Default.aspx).

## Funding

This research was funded by the National Cattlemen’s Beef Association, a contractor to the Beef Checkoff. The funder has no role in the research design, project execution, data interpretation, or manuscript preparation. The funder has no publication restrictions.

## Conflict of interest

KSP received a grant from the National Cattlemen’s Beef Association to conduct the research. KF and VLF at Nutrition Impact LLC perform consulting and database analyses for various food and beverage companies and related entities.
