# Supplementary material for: The contribution of beef consumed as part of the National School Lunch Program to nutrient intake and adequacy: a National Health and Nutrition Examination Survey 2003–2018 Analysis
Source: Curr Dev Nutr. 2025 Nov 24;9(12):107603. doi: 10.1016/j.cdnut.2025.107603 (PMC12799504; doi:10.1016/j.cdnut.2025.107603)
Supplement: multimedia component 1 [file mmc1.docx]

# **Supplementary Material**

## Supplementary Table 1: Median total daily beef intake and school lunch beef intake by beef consumption at school lunch status

|  | **Total daily beef intake (g)** | | | | | | **NSLP beef intake (g)** |
| --- | --- | --- | --- | --- | --- | --- | --- |
|  | NSLP beef non-consumers | | | | NSLP beef consumers | | |
| Age (years) & Gender Categories | n | All | n | Beef consumers only | n |  |  |
| 5-8 | 624 | 0 (0, 26) | 252 | 41 (13, 68) | 367 | 35 (11, 57) | 14 (5, 41) |
| Male | 326 | 0 (0, 26) | 139 | 33 (11, 67) | 194 | 35 (12, 63) | 13 (6, 38) |
| Female | 298 | 0 (0, 24) | 113 | 44 (20, 76) | 173 | 26 (9, 56) | 14 (3, 41) |
| 9-13 | 804 | 0 (0, 39) | 365 | 42 (16, 83) | 490 | 43 (13, 92) | 19 (5, 41) |
| Male | 409 | 0 (0, 43) | 184 | 58 (21, 111) | 252 | 45 (14, 115) | 18 (4, 41) |
| Female | 395 | 0 (0, 30) | 181 | 39 (14, 61) | 238 | 41 (13, 69) | 19 (6, 41) |
| 14-18 | 465 | 0 (0, 53) | 211 | 53 (30, 102) | 296 | 47 (12, 90) | 8 (4, 41) |
| Male | 237 | 1 (0, 84) | 126 | 84 (41, 127) | 172 | 64 (12, 107) | 8 (5, 41) |
| Female | 228 | 0 (0, 31) | 85 | 35 (18, 54) | 124 | 38 (12, 66) | 6 (3, 38) |
| 5-18 | 1893 | 0 (0, 39) | 828 | 45 (18, 84) | 1153 | 41 (13, 73) | 14 (5, 41) |
| Male | 972 | 0 (0, 52) | 449 | 56 (19, 102) | 618 | 44 (13, 93) | 14 (5, 41) |
| Female | 921 | 0 (0, 31) | 379 | 39 (16, 62) | 35 | 38 (12,63) | 15 (3, 41) |
| Food Security Status | | | | | | | |
| Food secure | 1578 | 0 (0, 40) | 691 | 44 (18, 84) | 939 | 44 (13, 73) | 14 (5, 41) |
| Low food security | 223 | 0 (0, 29) | 105 | 31 (13, 71) | 171 | 22 (6, 59) | 12 (4, 30) |
| Very low food security | 51 | 0 (0, 48) | 16 | 59 (47, 62) | 19 | 84 (20, 167) | 22 (5, 60) |

Data presented as median (interquartile range): NSLP National School Lunch Program

## Supplementary Table 2: Top contributors to beef intake in school lunch consumers of beef 5-18 y

| **Food Category** | **n consumers** | **Mean intake ± SEM^1^** |
| --- | --- | --- |
| Stir-fry and soy-based sauce mix | 2 | 76 |
| Meat mixed dishes | 105 | 59 ± 4.5 |
| Ground beef | 3 | 58 |
| Macaroni and cheese | 5 | 53 |
| Burritos and tacos | 105 | 51 ± 2.2 |
| Beef, excludes ground | 17 | 45 |
| Burgers | 54 | 42 ± 1.0 |
| Frankfurter sandwiches | 144 | 39 ± 4.1 |
| Nachos | 64 | 39 ± 1.5 |
| Turnovers and other grain-based | 19 | 32 ± 6.2 |
| Pasta mixed dishes, excludes macaroni & cheese | 89 | 31 ± 1.2 |
| Meat and BBQ sandwiches | 1 | 26 |
| Other Mexican mixed dishes | 12 | 19 ± 1.3 |
| Deli and cured meat sandwiches | 1 | 14 |
| Rice mixed dishes | 1 | 14 |
| Soups | 2 | 8.1 |
| Pasta sauces, tomato-based | 1 | 7.5 |
| Pizza | 564 | 5.6 ± 0.6 |

^1^SEM is not presented when the n is too small for SEM calculation; SEM standard error of the mean

## Supplementary Table 3: Total daily food group intake for NSLP consumers 5-18 y who consumed beef at school lunch compared to those who did not consume beef at school lunch

| **Food group** | **Beef consumers**  Mean ± SEM | **Beef non-consumers**  Mean ± SEM | **Beef consumers vs. non-consumers**  Mean difference ± SEM |
| --- | --- | --- | --- |
| Total vegetables (excludes legumes; cup-eq) | 1.07 ± 0.04 | 0.99 ± 0.03 | 0.08 ± 0.05 |
| Total fruits (cup-eq) | 1.11 ± 0.05 | 1.12 ± 0.05 | -0.01 ± 0.08 |
| Total grain (oz-eq) | 7.28 ± 0.10 | 7.26 ± 0.12 | 0.03 ± 0.14 |
| Whole grain (oz-eq) | 0.77 ± 0.06 | 0.82 ± 0.04 | -0.05 ± 0.06 |
| Refined grain (oz-eq) | 6.52 ± 0.11 | 6.44 ± 0.11 | 0.07 ± 0.14 |
| Total dairy (cup-eq) | 2.71 ± 0.06 | 2.57 ± 0.06 | 0.14 ± 0.09 |
| Nuts and seeds (oz-eq) | 0.16 ± 0.02 | 0.29 ± 0.04 | -0.12 ± 0.04* |
| Legumes (oz-eq) | 0.30 ± 0.03 | 0.30 ± 0.04 | 0.005 ± 0.05 |
| Soy products (excluding milk and mature soybeans; oz-eq) | 0.08 ± 0.01 | 0.07 ± 0.01 | 0.01 ± 0.02 |
| Beef, poultry, seafood (oz-eq) | 3.50 ± 0.11 | 3.62 ± 0.11 | -0.12 ± 0.15 |
| Meat (oz-eq) | 1.70 ± 0.11 | 1.03 ± 0.08 | 0.67 ± 0.13* |
| Cured meat (oz-eq) | 0.94 ± 0.07 | 0.63 ± 0.06 | 0.30 ± 0.08* |
| Organ meat (oz-eq) | 0.01 ± 0.01 | 0.01 ± 0.01 | 0.002 ± 0.01 |
| Poultry (oz-eq) | 0.73 ± 0.06 | 1.77 ± 0.08 | -1.05 ± 0.10* |
| Seafood high in n-3 fatty acids (oz-eq) | 0.01 ± 0.004 | 0.02 ± 0.01 | -0.02 ± 0.01* |
| Seafood low in n-3 fatty acids (oz-eq) | 0.12 ± 0.03 | 0.15 ± 0.02 | -0.03 ± 0.03 |
| Eggs and substitutes (oz-eq) | 0.24 ± 0.02 | 0.26 ± 0.02 | -0.02 ± 0.03 |
| Added sugars (tsp-eq) | 17.76 ± 0.44 | 18.83 ± 0.40 | -1.07 ± 0.56 |

cup-eq cup-equivalent; NSLP national school lunch program; oz-eq ounce-equivalent; SEM standard error of the mean; tsp-eq teaspoon equivalent; *p<0.05

## Supplementary Table 4: Nutrient intake of consumers of beef at school lunch, compared to non-consumers of beef at school lunch, by age category

| Nutrient | 5-8 y | | | 9-13 y | | | 14-18y | | |
| --- | --- | --- | --- | --- | --- | --- | --- | --- | --- |
|  | Beef consumer n=367 | Beef non-consumer n=624 | Difference | Beef consumer n=490 | Beef non-consumer n=804 | Difference | Beef consumer n=296 | Beef non-consumer n=465 | Difference |
| **Energy, kcal** | | | | | | | | | |
| Male | 1988 ± 58 | 1978 ± 41 | 10 ± 70 | 2315 ± 114 | 2067 ± 48 | 248 ± 119* | 2486 ± 114 | 2566 ± 104 | -80 ± 149 |
| Female | 1834 ± 49 | 1809 ± 46 | 26 ± 65 | 1979 ± 69 | 1963 ± 58 | 16 ± 93 | 1736 ± 121 | 1847 ± 68 | -111 ± 148 |
| **Protein, g** |  |  |  |  |  |  |  |  |  |
| Male | 71.0 ± 1.9 | 72.6 ± 1.4 | -1.6 ± 2.2 | 82.4 ± 2.1 | 80.2 ± 1.3 | 2.3 ± 2.3 | 98.9 ± 3.3 | 90.9 ± 2.5 | 8.0 ± 4.2 |
| Female | 62.0 ± 1.7 | 65.7 ± 1.2 | -3.6 ± 2.1 | 71.0 ± 1.3 | 70.0 ± 1.3 | 1.1 ± 1.9 | 66.1 ± 1.8 | 68.1 ± 2.4 | -2.1 ± 2.7 |
| **Total Fat, g** |  |  |  |  |  |  |  |  |  |
| Male | 74.1 ± 1.1 | 73.9 ± 0.9 | 0.2 ± 1.5 | 81.1 ± 1.5 | 79.0 ± 0.9 | 2.2 ± 1.6 | 96.5 ± 1.8 | 89.5 ± 2.7 | 7.0 ± 3.0* |
| Female | 67.0 ± 1.7 | 69.8 ± 0.9 | -2.8 ± 2.0 | 73.7 ± 1.5 | 71.7 ± 0.9 | 1.9 ± 1.8 | 77.7 ± 2.1 | 73.1 ± 1.5 | 4.6 ± 2.4 |
| **Saturated fat, g** |  |  |  |  |  |  |  |  |  |
| Male | 27.1 ± 0.7 | 27.2 ± 0.4 | -0.1 ± 0.8 | 30.6 ± 0.8 | 27.5 ± 0.5 | 3.1 ± 1.0* | 36.2 ± 0.9 | 30.8 ± 1.4 | 5.4 ± 1.7* |
| Female | 24.0 ± 0.8 | 24.5 ± 0.5 | -0.4 ± 0.9 | 26.6 ± 0.7 | 24.5 ± 0.6 | 2.1 ± 0.9* | 28.0 ± 0.8 | 24.2 ± 0.6 | 3.8 ± 1.0* |
| **Carbohydrate, g** |  |  |  |  |  |  |  |  |  |
| Male | 274 ± 2.8 | 273 ± 2.8 | 1.3 ± 4.0 | 277 ± 4.2 | 285 ± 2.8 | -7.8 ± 5.0 | 311 ± 5.9 | 337 ± 6.8 | -25.8 ± 9.0* |
| Female | 255 ± 5.0 | 244 ± 2.2 | 10 ± 5.7 | 252 ± 4.1 | 258 ± 2.7 | -6.0 ± 4.7 | 237 ± 4.9 | 247 ± 4.8 | -10.4 ± 5.9 |
| **Dietary fiber, g** |  |  |  |  |  |  |  |  |  |
| Male | 15.3 ± 0.5 | 14.1 ± 0.4 | 1.2 ± 0.6 | 15.5 ± 0.6 | 16.4 ± 0.5 | -0.9 ± 0.8 | 16.8 ± 0.7 | 16.2 ± 0.5 | 0.6 ± 0.8 |
| Female | 14.5 ± 0.4 | 14.1 ± 0.3 | 0.3 ± 0.5 | 14.8 ± 0.6 | 14.5 ± 0.4 | 0.4 ± 0.7 | 12.7 ± 0.6 | 14.5 ± 0.6 | -1.8 ± 0.8* |
| **Calcium, mg** | | | | | | | | | |
| Male | 1200 ± 39 | 1247 ± 33 | -47 ± 48 | 1261 ± 39 | 1230 ± 27 | 31 ± 47 | 1403 ± 62 | 1177 ± 61 | 226 ± 90* |
| Female | 1044 ± 39 | 1069 ± 33 | -24 ± 51 | 1089 ± 27 | 1064 ± 30 | 26 ± 40 | 1013 ± 45 | 963 ± 38 | 49 ± 55 |
| **Sodium, mg** |  |  |  |  |  |  |  |  |  |
| Male | 3192 ± 57 | 3006 ± 55 | 186 ± 81* | 3505 ± 63 | 3539 ± 66 | -34 ± 92 | 4093 ± 98 | 4031 ± 82 | 62 ± 127 |
| Female | 2866 ± 62 | 2827 ± 70 | 39.0 ± 89 | 3065 ± 56 | 3083 ± 47 | -19 ± 70 | 3221 ± 109 | 3067 ± 94 | 154 ± 113 |
| **Potassium, mg** |  |  |  |  |  |  |  |  |  |
| Male | 2421 ± 58 | 2502 ± 53 | -81 ± 79 | 2622 ± 73 | 2518 ± 50 | 103 ± 89 | 2862 ± 79 | 2731 ± 80 | 131 ± 111 |
| Female | 2206 ± 53 | 2229 ± 42 | -23 ± 58 | 2331 ± 47 | 2210 ± 43 | 121 ± 64 | 1980 ± 77 | 2028 ± 100 | -49 ± 104 |
| **Copper, mg** | | | | | | | | | |
| Male | 1.1 ± 0.04 | 1.1 ± 0.1 | -0.1 ± 0.1 | 1.1 ± 0.03 | 1.1 ± 0.02 | -0.01 ± 0.04 | 1.2 ± 0.05 | 1.2 ± 0.03 | 0.0 ± 0.1 |
| Female | 0.9 ± 0.02 | 0.9 ± 0.02 | 0.0 ± 0.02 | 1.0 ± 0.03 | 1.0 ± 0.02 | 0.04 ± 0.03 | 0.9 ± 0.03 | 1.0 ± 0.03 | -0.1 ± 0.03* |
| **Folate, DFE µg** | | | | | | | | | |
| Male | 567 ± 26 | 581 ± 22 | -14 ± 34 | 606 ± 34 | 568 ± 20 | 38 ± 41 | 650 ± 39 | 580 ± 31 | 70 ± 50 |
| Female | 503 ± 20 | 530 ± 36 | -27 ± 32 | 510 ± 26 | 504 ± 20 | 6 ± 32 | 448 ± 31 | 477 ± 26 | -29 ± 37 |
| **Iron, mg** | | | | | | | | | |
| Male | 15.6 ± 0.6 | 14.4 ± 0.3 | 1.1 ± 0.7 | 16.2 ± 0.5 | 16.2 ± 0.4 | 0.1 ± 0.6 | 17.0 ± 0.6 | 16.1 ± 0.4 | 0.9 ± 0.7 |
| Female | 14.3 ± 0.5 | 13.6 ± 0.5 | 0.6 ± 0.6 | 13.6 ± 0.4 | 13.9 ± 0.4 | -0.3 ± 0.6 | 12.2 ± 0.5 | 13.6 ± 0.6 | -1.4 ± 0.8 |
| **Magnesium, mg** | | | | | | | | | |
| Male | 240 ± 4.7 | 248 ± 3.9 | -7.9 ± 5.8 | 253 ± 5.2 | 264 ± 4.4 | -12 ± 7.3 | 295 ± 10 | 288 ± 6.7 | 6.8 ± 12 |
| Female | 224 ± 4.5 | 227 ± 3.6 | -3.1 ± 5.1 | 233 ± 4.6 | 234 ± 5.7 | -1.5 ± 7.2 | 210 ± 5.9 | 239 ± 7.1 | -29 ± 8.2* |
| **Niacin, mg** | | | | | | | | | |
| Male | 21.2 ± 0.8 | 21.8 ± 0.4 | -0.6 ± 0.9 | 24.1 ± 1.0 | 25.0 ± 0.7 | -0.9 ± 1.3 | 27.3 ± 0.8 | 28.1 ± 0.9 | -0.8 ± 1.3 |
| Female | 19.7 ± 0.7 | 19.8 ± 0.6 | -0.1 ± 0.9 | 19.4 ± 0.6 | 21.0 ± 0.5 | -1.6 ± 0.8* | 17.7 ± 0.8 | 22.2 ± 1.1 | -4.5 ± 1.1* |
| **Phosphorus, mg** | | | | | | | | | |
| Male | 1377 ± 33 | 1427 ± 26 | -51 ± 38 | 1492 ± 27 | 1482 ± 19 | 10 ± 33 | 1756 ± 48 | 1575 ± 33 | 181 ± 59* |
| Female | 1237 ± 35 | 1301 ± 21 | -64 ± 38 | 1307 ± 18 | 1311 ± 25 | -3 ± 33 | 1214 ± 30 | 1233 ± 33 | -19 ± 41 |
| **Selenium, µg** | | | | | | | | | |
| Male | 93.8 ± 3.0 | 95.8 ± 2.0 | -2.0 ± 3.8 | 112 ± 2.4 | 108 ± 1.9 | 3.4 ± 2.8 | 136 ± 4.9 | 126 ± 3.7 | 10.6 ± 5.9 |
| Female | 84.5 ± 2.0 | 85.6 ± 1.9 | -1.2 ± 2.8 | 96.2 ± 2.3 | 93.9 ± 2.1 | 2.3 ± 3.2 | 88.0 ± 2.6 | 97.0 ± 2.5 | -9.0 ± 3.2* |
| **Thiamin, mg** | | | | | | | | | |
| Male | 1.6 ± 0.1 | 1.7 ± 0.04 | -0.05 ± 0.1 | 1.8 ± 0.1 | 1.8 ± 0.04 | 0.04 ±0.08 | 2.2 ± 0.1 | 1.9 ± 0.1 | 0.2 ± 0.1 |
| Female | 1.5 ± 0.05 | 1.5 ± 0.04 | 0.02 ± 0.1 | 1.5 ± 0.04 | 1.5 ± 0.03 | -0.02 ± 0.05 | 1.4 ± 0.1 | 1.5 ± 0.1 | -0.1 ± 0.1 |
| **Riboflavin, mg** | | | | | | | | | |
| Male | 2.3 ± 0.1 | 2.5 ± 0.1 | -0.1 ± 0.1 | 2.4 ± 0.1 | 2.4 ± 0.05 | 0.1 ± 0.1 | 2.6 ± 0.08 | 2.3 ± 0.1 | 0.3 ± 0.1 |
| Female | 2.0 ± 0.1 | 2.1 ± 0.05 | -0.03 ± 0.1 | 2.0 ± 0.1 | 2.0 ± 0.05 | 0.01 ± 0.07 | 1.7 ± 0.1 | 1.8 ± 0.1 | -0.1 ± 0.1 |
| **Vitamin A, retinol equivalents** | | | | | | | | | |
| Male | 760 ± 49 | 799 ± 55 | -39 ± 75 | 724 ± 37 | 727 ± 26 | -3.0 ± 43 | 662 ± 34 | 638 ± 58 | 24 ± 68 |
| Female | 656 ± 41 | 581 ± 24 | 74 ± 50 | 597 ± 36 | 604 ± 24 | -6.6 ± 42 | 491 ± 32 | 549 ± 46 | -58 ± 50 |
| **Vitamin B6, mg** | | | | | | | | | |
| Male | 1.7 ± 0.1 | 1.8 ± 0.05 | -0.1 ± 0.1 | 2.0 ± 0.1 | 2.0 ± 0.1 | -0.03 ± 0.1 | 2.1 ± 0.1 | 2.1 ± 0.1 | -0.02 ± 0.1 |
| Female | 1.6 ± 0.1 | 1.6 ± 0.1 | -0.02 ± 0.1 | 1.5 ± 0.1 | 1.6 ± 0.1 | -0.1 ±0.1 | 1.4 ± 0.1 | 1.7 ± 0.1 | -0.3 ± 0.1* |
| **Vitamin B12, µg** | | | | | | | | | |
| Male | 5.7 ± 0.2 | 6.0 ± 0.5 | -0.3 ± 0.6 | 6.7 ± 0.4 | 5.6 ± 0.2 | 1.0 ± 0.5* | 6.7 ± 0.4 | 5.4 ± 0.4 | 1.3 ± 0.5* |
| Female | 5.0 ± 0.2 | 4.7 ± 0.2 | 0.3 ± 0.3 | 5.1 ± 0.2 | 4.4 ± 0.2 | 0.6 ± 0.3* | 4.1 ± 0.2 | 3.9 ± 0.3 | 0.2 ± 0.3 |
| **Vitamin C, mg** | | | | | | | | | |
| Male | 85.9 ± 7.4 | 86.9 ± 4.3 | -0.9 ± 8.9 | 78.9 ± 6.2 | 79.8 ± 4.2 | -0.9 ± 7.4 | 90.5 ± 12.4 | 112 ± 25.1 | -21.8 ± 26.9 |
| Female | 82.6 ± 6.6 | 71.6 ± 5.1 | 11.0 ± 8.6 | 76.0 ± 5.0 | 73.5 ± 4.8 | 2.6 ± 7.1 | 69.2 ± 9.0 | 70.3 ± 7.8 | -1.1 ± 11.2 |
| **Vitamin D (D2+D3), µg** | | | | | | | | | |
| Male | 7.6 ± 0.3 | 8.0 ± 0.3 | -0.4 ± 0.4 | 7.4 ± 0.4 | 7.0 ± 0.3 | 0.4 ± 0.5 | 7.5 ± 0.5 | 6.8 ± 0.6 | 0.7 ± 0.8 |
| Female | 6.1 ± 0.4 | 6.6 ± 0.2 | -0.5 ± 0.4 | 6.1 ± 0.3 | 5.7 ± 0.3 | 0.4 ± 0.3 | 3.9 ± 0.4 | 4.3 ± 0.4 | -0.4 ± 0.5 |
| **Vitamin E (alpha tocopherol), mg** | | | | | | | | | |
| Male | 5.9 ± 0.2 | 6.7 ± 0.4 | -0.8 ± 0.4 | 6.8 ± 0.4 | 7.2 ± 0.2 | -0.4 ± 0.4 | 7.1 ± 0.3 | 7.9 ± 0.3 | -0.8 ± 0.4 |
| Female | 6.3 ± 0.3 | 5.9 ± 0.2 | 0.4 ± 0.4 | 6.1 ± 0.2 | 6.6 ± 0.2 | -0.5 ± 0.3 | 6.2 ± 0.3 | 7.0 ± 0.4 | -0.8 ± 0.4* |
| **Zinc, mg** | | | | | | | | | |
| Male | 11.3 ± 0.4 | 11.3 ± 0.3 | 0.1 ± 0.5 | 13.9 ± 0.6 | 12.0 ± 0.4 | 1.9 ± 0.7* | 14.5 ± 0.7 | 12.5 ± 0.6 | 2.0 ± 0.9* |
| Female | 9.8 ± 0.4 | 9.7 ± 0.3 | 0.1 ± 0.4 | 10.8 ± 0.3 | 9.6 ± 0.3 | 1.2 ± 0.4* | 10.0 ± 0.4 | 9.4 ± 0.6 | 0.6 ± 0.6 |
| **Choline, mg** | | | | | | | | | |
| Male | 244 ± 7.7^1^ | 269 ± 9.4^2^ | -25 ± 11.6* | 295 ± 14.4^3^ | 262 ± 6.3^4^ | 33.4 ± 15.2* | 341 ± 14^5^ | 318 ± 15.2^6^ | 22.3 ± 20.4 |
| Female | 220 ± 6.3^7^ | 236 ± 7.1^8^ | -16 ± 9.8 | 251 ± 6.6^9^ | 236 ± 6.0^10^ | 15.4 ± 9.4 | 215 ± 12.7^11^ | 213 ± 12.7^12^ | 2.4 ± 13.8 |

Data presented as least squares mean ± standard error; DFE Dietary Folate Equivalents

*p<0.05; presented n are unweighted; ^1^n=171; ^2^n=292; ^3^n=221; ^4^n=362; ^5^n=146; ^6^n=197; ^7^n=154; ^8^n=272; ^9^n=207; ^10^n=336; ^11^n=103; ^12^n=198

## Supplementary Table 5: Nutrient intake of consumers of beef at school lunch, compared to non-consumers of beef at school lunch, by food security status

| Nutrient | Food secure | | | Low food security | | | Very low food security | | |
| --- | --- | --- | --- | --- | --- | --- | --- | --- | --- |
|  | Beef consumer n=939 | Beef non-consumer n=1,578 | Difference | Beef consumer n=171 | Beef non-consumer n=223 | Difference | Beef consumer n=19 | Beef non-consumer n=51 | Difference |
| **Energy, kcal** | 2086 ± 39 | 2044 ± 26 | 42 ± 48 | 1906 ± 84 | 2019 ± 91 | -113 ± 122 | 2185 ± 156 | 1821 ± 151 | 364 ± 214 |
| **Protein, g** | 75.6 ± 1.1 | 74.5 ± 0.8 | 1.1 ±1.3 | 74.3 ± 1.5 | 75.8 ± 1.9 | -1.5 ± 2.4 | 78.7 ± 4.8 | 70.4 ± 2.7 | 8.3 ± 5.8 |
| **Total Fat, g** | 78.1 ± 0.9 | 75.5 ± 0.7 | 2.6 ± 1.1* | 74.9 ± 1.5 | 76.6 ± 1.5 | -1.7 ± 1.9 | 71.4 ± 3.2 | 75.1 ± 2.8 | -3.7 ± 4.2 |
| **Saturated fat, g** | 28.8 ± 0.5 | 26.2 ± 0.3 | 2.6 ± 0.6* | 26.4 ± 0.8 | 27.1 ± 0.7 | -0.7 ± 0.9 | 28.6 ± 1.6 | 27.4 ± 1.3 | 1.2 ± 1.9 |
| **Carbohydrate, g** | 267 ± 2.6 | 275 ± 1.9 | -7.7 ± 3.3* | 268 ± 3.6 | 264 ± 4.8 | 4.2 ± 5.7 | 252 ± 10.7 | 254 ± 8.7 | -1.9 ± 14.2 |
| **Dietary fiber, g** | 15.1 ± 0.3 | 14.9 ± 0.2 | 0.2 ± 0.3 | 14.9 ± 0.4 | 16.3 ± 0.6 | -1.4 ± 0.7 | 12.2 ± 1.7 | 13.9 ± 1.0 | -1.7 ± 2.0 |
| **Calcium, mg** | 1184 ± 20 | 1130 ± 21 | 54 ± 30 | 1116 ± 52 | 1176 ± 37 | -60 ± 60 | 1133 ± 60 | 1133 ± 42 | 0.1 ± 74 |
| **Sodium, mg** | 3313 ± 34 | 3256 ± 39 | 57 ± 44 | 3320 ± 54 | 3279 ± 76 | 42 ± 95 | 3100 ± 143 | 2830 ± 101 | 270 ± 204 |
| **Potassium, mg** | 2417 ± 31 | 2371 ± 29 | 46 ± 36 | 2470 ± 79 | 2521 ± 61 | -51 ± 98 | 2289 ± 96 | 2224 ± 100 | 65 ± 135 |
| **Copper, mg** | 1.0 ± 0.02 | 1.1 ± 0.02 | -0.02 ± 0.03 | 1.0 ± 0.03 | 1.1 ± 0.02 | -0.01 ± 0.04 | 0.8 ± 0.1 | 0.9 ± 0.1 | -0.1 ± 0.1 |
| **Folate, DFE µg** | 556 ± 14 | 542 ± 12 | 14 ± 19 | 535 ± 30 | 545 ± 26 | -11 ± 37 | 608 ± 90 | 517 ± 48 | 91 ± 101 |
| **Iron, mg** | 15.1 ± 0.3 | 14.7 ± 0.2 | 0.4 ± 0.3 | 14.1 ± 0.4 | 14.5 ± 0.5 | -0.4 ± 0.6 | 14.6 ± 1.0 | 13.4 ± 1.0 | 1.2 ± 1.3 |
| **Magnesium, mg** | 244 ± 3.0 | 250 ± 2.8 | -5.6 ± 3.9 | 239 ± 5.2 | 257 ± 6.5 | -18.7 ± 8.5* | 234 ± 14.1 | 228 ± 6.2 | 6.0 ± 15.2 |
| **Niacin, mg** | 21.8 ± 0.4 | 23.1 ± 0.3 | -1.2 ± 0.5* | 20.7 ± 0.7 | 22.3 ± 0.5 | -1.6 ± 0.8 | 20.9 ± 1.7 | 22.1 ± 1.1 | -1.2 ± 2.1 |
| **Phosphorus, mg** | 1406 ± 18 | 1392 ± 14 | 14 ± 22 | 1377 ± 26 | 1422 ± 24 | -45 ± 34 | 1395 ± 61 | 1295 ± 37 | 101 ± 75 |
| **Selenium, µg** | 102 ± 1.5 | 101 ± 1.2 | 1.3 ± 1.8 | 104 ± 3.9 | 99.1 ± 2.7 | 5.0 ± 4.7 | 99.0 ± 6.2 | 93.1 ± 3.5 | 5.9 ± 7.7 |
| **Thiamin, mg** | 1.7 ± 0.03 | 1.7 ± 0.02 | 0.04 ± 0.03 | 1.7 ± 0.1 | 1.6 ± 0.05 | 0.1 ± 0.1 | 1.5 ± 0.1 | 1.6 ± 0.1 | -0.1 ± 0.1 |
| **Riboflavin, mg** | 2.2 ± 0.03 | 2.2 ± 0.03 | 0.1 ± 0.05 | 2.0 ± 0.1 | 2.2 ± 0.1 | -0.2 ± 0.1 | 2.3 ± 0.2 | 2.2 ± 0.1 | 0.04 ± 0.2 |
| **Vitamin A, retinol equivalents** | 678 ± 20 | 654 ± 21 | 23 ± 27 | 561 ± 29 | 684 ± 43 | -123 ± 50* | 488 ± 45 | 711 ± 41 | -223 ± 62* |
| **Vitamin B6, mg** | 1.7 ± 0.05 | 1.8 ± 0.03 | -0.1 ± 0.1 | 1.6 ± 0.1 | 1.8 ± 0.1 | -0.2 ± 0.1 | 1.7 ± 0.2 | 1.8 ± 0.1 | -0.1 ± 0.2 |
| **Vitamin B12, µg** | 5.8 ± 0.2 | 5.1 ± 0.2 | 0.7 ± 0.2* | 5.0 ± 0.2 | 5.3 ± 0.3 | -0.3 ± 0.3 | 6.2 ± 0.7 | 4.9 ± 0.4 | 1.3 ± 0.8 |
| **Vitamin C, mg** | 76.8 ± 3.4 | 81.4 ± 5.0 | -4.6 ± 6.1 | 91.7 ± 12 | 89.4 ± 7.3 | 2.3 ± 14 | 45.1 ± 9.2 | 72.8 ± 9.3 | -28 ± 11* |
| **Vitamin D (D2+D3), µg** | 6.7 ± 0.2 | 6.4 ± 0.2 | 0.2 ± 0.2 | 6.3 ± 0.3 | 7.1 ± 0.4 | -0.7 ± 0.5 | 7.3 ± 0.9 | 6.9 ± 0.4 | 0.4 ± 1.0 |
| **Vitamin E (alpha tocopherol), mg** | 6.4 ± 0.1 | 6.8 ± 0.1 | -0.5 ± 0.2* | 6.2 ± 0.2 | 6.9 ± 0.3 | -0.7 ± 0.4* | 5.4 ± 0.7 | 7.0 ± 0.6 | -1.6 ± 0.9 |
| **Zinc, mg** | 12.0 ± 0.3 | 10.8 ± 0.2 | 1.2 ± 0.3* | 11.0 ± 0.4 | 10.8 ± 0.4 | 0.2 ± 0.6 | 14.1 ± 1.7 | 10.3 ± 0.5 | 3.8 ± 1.7* |
| **Choline, mg** | 265 ± 5.3^1^ | 252 ± 4.6^2^ | 12.6 ± 5.9* | 255 ± 9.6^3^ | 265 ± 10.1^4^ | -9.2 ± 14.9 | 244 ± 25.1^5^ | 256 ± 18.1^6^ | -12.4 ± 33.8 |

Data presented as least squares mean ± standard error; DFE Dietary Folate Equivalents

*p<0.05; ^1^n=821; ^2^n=1,395; ^3^n=146; ^4^n=184; ^5^n=15; ^6^n=48

## Supplementary Table 6: The percentage of consumers of beef at school lunch, compared to non-consumers of beef at school lunch, who have a nutrient intake below the estimated average requirement (EAR)/above the Adequate Intake (AI) level by gender- age category

| Nutrient | 5-8 y | | | 9-13 y | | | 14-18y | | |
| --- | --- | --- | --- | --- | --- | --- | --- | --- | --- |
|  | Beef consumer n=367 | Beef non-consumer n=624 | Difference | Beef consumer n=490 | Beef non-consumer n=804 | Difference | Beef consumer n=296 | Beef non-consumer n=465 | Difference |
| **Calcium, mg** | | | | | | | | | |
| Male | 7.14 ± 2.52 | 6.50 ± 1.98 | 0.63 ± 3.21 | 29.64 ± 4.75 | 38.02 ± 3.45 | -8.38 ± 5.87 | 35.70 ± 6.43 | 48.47 ± 5.67 | -12.77 ± 8.57 |
| Female | 21.13 ± 4.97 | 17.59 ± 3.28 | 3.53 ± 5.96 | 56.13 ± 4.68 | 59.95 ± 3.89 | -3.82 ± 6.09 | 63.91 ± 6.79 | 69.59 ± 4.98 | -5.68 ± 8.42 |
| **Choline^1^** | | | | | | | | | |
| Male | 67.06 ± 6.35^2^ | 61.71 ± 3.60^3^ | 5.35 ± 7.30 | 18.33 ± 6.30^4^ | 7.71 ± 2.18^5^ | 10.62 ± 6.67 | 0.49 ± 0.66^6^ | 0.18 ± 0.17^7^ | 0.32 ± 0.69 |
| Female | 30.28 ± 5.35^8^ | 33.58 ± 3.93^9^ | -3.30 ± 6.64 | 3.53 ± 2.06^10^ | 1.51 ± 0.64^11^ | 2.02 ± 2.16 | 1.75 ± 1.61^12^ | 1.45 ± 0.77^13^ | 0.30 ± 1.78 |
| **Copper, mg** | | | | | | | | | |
| Male | 0.00 ± 0.01 | 0.001 ± 0.004 | -0.001 ± 0.01 | 0.07 ± 0.16 | 0.55 ± 0.33 | -0.48 ± 0.37 | 1.89 ± 1.22 | 4.21 ± 1.51 | -2.31 ± 1.94 |
| Female | 0.01 ± 0.02 | 0.02 ± 0.02 | -0.003 ± 0.03 | 0.94 ± 0.69 | 1.73 ± 0.72 | -0.79 ± 1.00 | 8.16 ± 3.88 | 8.97 ± 2.74 | -0.80 ± 4.75 |
| **Folate, DFE µg** | | | | | | | | | |
| Male | 0.03 ± 0.04 | 0.02 ± 0.02 | 0.02 ± 0.05 | 0.46 ± 0.41 | 0.65 ± 0.37 | -0.18 ± 0.55 | 6.85 ± 2.94 | 5.70 ± 2.04 | 1.15 ± 3.58 |
| Female | 0.14 ± 0.19 | 0.03 ± 0.06 | 0.10 ± 0.20 | 2.48 ± 1.45 | 2.03 ± 1.04 | 0.45 ± 1.78 | 19.39 ± 5.74 | 12.16 ± 3.55 | 7.23 ± 6.75 |
| **Iron, mg** | | | | | | | | | |
| Male | 0.08 ± 0.07 | 0.28 ± 0.13 | -0.20 ± 0.15 | 0.00 ± 0.00 | 0.00 ± 0.03 | 0.00 ± 0.03 | 1.04 ± 0.65 | 1.80 ± 0.81 | -0.76 ± 1.04 |
| Female | 0.68 ± 0.27 | 0.99 ± 0.31 | -0.31 ± 0.41 | 0.27 ± 0.22 | 0.31 ± 0.23 | -0.04 ± 0.32 | 10.94 ± 2.29 | 9.49 ± 2.07 | 1.44 ± 3.09 |
| **Magnesium, mg** | | | | | | | | | |
| Male | 0.04 ± 0.07 | 0.05 ± 0.10 | -0.01 ± 0.12 | 10.29 ± 3.45 | 14.84 ± 2.79 | -4.55 ± 4.44 | 88.48 ± 3.98 | 87.25 ± 3.28 | 1.23 ± 5.15 |
| Female | 0.39 ± 0.46 | 0.35 ± 0.24 | 0.04 ± 0.52 | 31.99 ± 5.49 | 28.61 ± 3.19 | 3.39 ± 6.35 | 91.46 ± 2.96 | 85.01 ± 4.13 | 6.45 ± 5.09 |
| **Niacin, mg** | | | | | | | | | |
| Male | 0.00 ± 0.00 | 0.00 ± 0.001 | 0.00 ± 0.001 | 0.002 ± 0.02 | 0.01 ± 0.02 | -0.01 ± 0.03 | 0.24 ± 0.27 | 0.05 ± 0.14 | 0.19 ± 0.30 |
| Female | 0.01 ± 0.02 | 0.001 ± 0.01 | 0.01 ± 0.02 | 0.08 ± 0.28 | 0.12 ± 0.15 | -0.04 ± 0.32 | 1.72 ± 1.15 | 0.34 ± 0.36 | 1.37 ± 1.21 |
| **Phosphorus, mg** | | | | | | | | | |
| Male | 0.00 ± 0.002 | 0.00 ± 0.002 | 0.00 ± 0.003 | 5.28 ± 2.19 | 7.58 ± 1.92 | -2.31 ± 2.91 | 4.77 ± 2.08 | 7.24 ± 2.20 | -2.47 ± 3.03 |
| Female | 0.00 ± 0.02 | 0.01 ± 0.01 | -0.01 ± 0.03 | 24.30 ± 4.82 | 21.85 ± 3.19 | 2.44 ± 5.78 | 23.44 ± 5.15 | 20.54 ± 3.63 | 2.91 ± 6.30 |
| **Potassium^1^** | | | | | | | | | |
| Male | 66.74 ± 5.04 | 65.61 ± 3.45 | 1.13 ± 6.11 | 62.96 ± 5.68 | 48.99 ± 3.58 | 13.96 ± 6.72* | 20.58 ± 6.38 | 18.10 ± 4.11 | 2.48 ± 7.59 |
| Female | 34.06 ± 4.78 | 39.38 ± 4.30 | -5.32 ± 6.43 | 46.41 ± 5.33 | 36.62 ± 3.72 | 9.79 ± 6.50 | 35.13 ± 7.24 | 34.86 ± 5.27 | 0.27 ± 8.96 |
| **Riboflavin, mg** | | | | | | | | | |
| Male | 0.00 ± 0.004 | 0.00 ± 0.001 | 0.00 ± 0.004 | 0.02 ± 0.04 | 0.02 ± 0.04 | -0.01 ± 0.06 | 0.85 ± 0.50 | 1.17 ± 0.66 | -0.33 ± 0.82 |
| Female | 0.002 ± 0.03 | 0.00 ± 0.01 | 0.002 ± 0.03 | 0.38 ± 0.32 | 0.31 ± 0.20 | 0.07 ± 0.38 | 1.94 ± 1.10 | 1.25 ± 0.72 | 0.69 ± 1.32 |
| **Selenium, µg** | | | | | | | | | |
| Male | 0.00 ± 0.001 | 0.001 ± 0.002 | -0.001 ± 0.002 | 0.00 ± 0.001 | 0.00 ± 0.004 | 0.00 ± 0.004 | 0.001 ± 0.02 | 0.004 ± 0.02 | -0.003 ± 0.03 |
| Female | 0.00 ± 0.005 | 0.00 ± 0.003 | 0.00 ± 0.01 | 0.001 ± 0.04 | 0.01 ± 0.03 | -0.01 ± 0.05 | 0.09 ± 0.19 | 0.04 ± 0.10 | 0.05 ± 0.22 |
| **Sodium^1^** | | | | | | | | | |
| Male | 100 ± 0.000 | 100 ± 0.004 | 0.00 ± 0.004 | 100 ± 0.000 | 100 ± 0.007 | 0.00 ± 0.007 | 100 ± 0.003 | 99.99 ± 0.02 | 0.01 ± 0.02 |
| Female | 100 ± 0.002 | 99.99 ± 0.02 | 0.009 ± 0.02 | 100 ± 0.004 | 99.97 ± 0.03 | 0.03 ± 0.03 | 100 ± 0.03 | 99.92 ± 0.12 | 0.08 ± 0.12 |
| **Thiamin, mg** | | | | | | | | | |
| Male | 0.00 ± 0.01 | 0.00 ± 0.01 | 0.00 ± 0.01 | 0.04 ± 0.06 | 0.05 ± 0.05 | -0.01 ± 0.08 | 1.65 ± 1.14 | 1.48 ± 0.71 | 0.17 ± 1.34 |
| Female | 0.08 ± 0.08 | 0.01 ± 0.03 | 0.07 ± 0.08 | 0.55 ± 0.44 | 0.35 ± 0.28 | 0.19 ± 0.52 | 4.77 ± 2.60 | 2.68 ± 1.16 | 2.09 ± 2.84 |
| **Vitamin A, retinol equivalents** | | | | | | | | | |
| Male | 0.15 ± 0.32 | 0.96 ± 0.48 | -0.80 ± 0.57 | 9.17 ± 3.06 | 13.61 ± 2.61 | -4.44 ± 4.02 | 57.56 ± 7.54 | 59.45 ± 6.88 | -1.90 ± 10.21 |
| Female | 1.23 ± 1.02 | 2.23 ± 1.28 | -1.00 ± 1.64 | 18.50 ± 5.13 | 20.13 ± 3.46 | -1.64 ± 6.19 | 50.57 ± 7.10 | 48.33 ± 7.03 | 2.24 ± 9.99 |
| **Vitamin B6, mg** | | | | | | | | | |
| Male | 0.00 ± 0.01 | 0.001 ± 0.01 | -0.001 ± 0.01 | 0.12 ± 0.26 | 0.26 ± 0.22 | -0.14 ± 0.35 | 5.58 ± 2.73 | 3.56 ± 1.58 | 2.02 ± 3.16 |
| Female | 0.03 ± 0.08 | 0.03 ± 0.06 | -0.004 ± 0.01 | 1.99 ± 1.33 | 1.60 ± 0.79 | 0.38 ± 1.54 | 14.74 ± 5.71 | 6.67 ± 2.42 | 8.06 ± 6.20 |
| **Vitamin B12, µg** | | | | | | | | | |
| Male | 0.00 ± 0.00 | 0.00 ± 0.01 | 0.00 ± 0.006 | 0.00 ± 0.01 | 0.07 ± 0.07 | -0.07 ± 0.07 | 0.07 ± 0.11 | 0.89 ± 0.66 | -0.82 ± 0.67 |
| Female | 0.00 ± 0.006 | 0.004 ± 0.04 | -0.004 ± 0.04 | 0.03 ± 0.09 | 0.41 ± 0.30 | -0.38 ± 0.31 | 0.51 ± 0.91 | 4.02 ± 1.77 | -3.51 ± 1.99 |
| **Vitamin C, mg** | | | | | | | | | |
| Male | 0.68 ± 0.52 | 0.94 ± 0.76 | -0.26 ± 0.92 | 8.80 ± 2.93 | 10.53 ± 3.86 | -1.74 ± 4.85 | 52.71 ± 7.18 | 43.65 ± 7.98 | 9.06 ± 10.74 |
| Female | 0.92 ± 0.69 | 1.92 ± 1.17 | -1.01 ± 1.36 | 10.31 ± 3.67 | 16.84 ± 4.33 | -6.53 ± 5.68 | 47.37 ± 8.09 | 46.51 ± 7.13 | 0.86 ± 10.78 |
| **Vitamin D (D2+D3), µg** | | | | | | | | | |
| Male | 75.4 ± 4.1 | 75.4 ± 3.0 | -0.05 ± 5.1 | 79.27 ± 4.41 | 84.36 ± 2.52 | -5.09 ± 5.08 | 90.83 ± 3.49 | 92.73 ± 2.28 | -1.89 ± 4.17 |
| Female | 91.7 ± 2.5 | 88.7 ± 2.1 | 2.9 ± 3.3 | 94.24 ± 2.16 | 94.01 ± 1.68 | 0.23 ± 2.73 | 97.43 ± 1.35 | 97.50 ± 1.06 | -0.07 ± 1.72 |
| **Vitamin E (alpha tocopherol), mg** | | | | | | | | | |
| Male | 41.1 ± 5.8 | 37.5 ± 4.3 | 3.6 ± 7.2 | 85.60 ± 4.27 | 79.70 ± 3.10 | 5.90 ± 5.28 | 99.09 ± 0.99 | 96.07 ± 1.84 | 3.01 ± 2.09 |
| Female | 62.9 ± 6.0 | 47.4 ± 4.5 | 15.5 ± 7.5* | 94.56 ± 2.57 | 86.45 ± 2.41 | 8.11 ± 3.53* | 99.63 ± 0.38 | 98.04 ± 1.09 | 1.60 ± 1.15 |
| **Zinc, mg** | | | | | | | | | |
| Male | 0.01 ± 0.01 | 0.01 ± 0.03 | -0.001 ± 0.03 | 0.54 ± 0.77 | 3.48 ± 1.33 | -2.94 ± 1.53 | 4.05 ± 2.37 | 11.21 ± 3.60 | -7.17 ± 4.31 |
| Female | 0.07 ± 0.17 | 0.16 ± 0.16 | -0.10 ± 0.23 | 6.39 ± 2.81 | 13.76 ± 2.90 | -7.37 ± 4.04 | 8.62 ± 3.25 | 15.74 ± 4.81 | -7.12 ± 5.81 |

Data are the percentage below the EAR unless otherwise stated; ^1^above the AI level; ^2^n=171; ^3^n=292; ^4^n=221; ^5^n=362; ^6^n=146; ^7^n=197; ^8^n=154; ^9^n=272; ^10^n=207; ^11^n=336; ^12^n=103; ^13^n=198; reduced n for choline was due to not having choline intake in early NHANES releases; AI adequate intake; DFE Dietary Folate Equivalents; EAR estimated average requirement; presented n are unweighted

*p<0.05
